# Physical Therapists’ Knowledge and Attitudes Regarding Artificial Intelligence Applications in Health Care and Rehabilitation: Cross-sectional Study

**DOI:** 10.2196/39565

**Published:** 2022-10-20

**Authors:** Mashael Alsobhi, Fayaz Khan, Mohamed Faisal Chevidikunnan, Reem Basuodan, Lama Shawli, Ziyad Neamatallah

**Affiliations:** 1 Department of Physical Therapy Faculty of Medical Rehabilitation Sciences King Abdulaziz University Jeddah Saudi Arabia; 2 Department of Rehabilitation Sciences College of Health and Rehabilitation Sciences Princess Nourah bint Abdulrahman University Riyadh Saudi Arabia; 3 Department of Occupational Therapy College of Applied Medical Sciences King Saud bin Abdulaziz University for Health Sciences Jeddah Saudi Arabia

**Keywords:** artificial intelligence, physical therapy, clinicians’ attitudes, health care, rehabilitation, digital health, machine learning, survey

## Abstract

**Background:**

The use of artificial intelligence (AI) in the field of rehabilitation is growing rapidly. Therefore, there is a need to understand how physical therapists (PTs) perceive AI technologies in clinical practice.

**Objective:**

This study aimed to investigate the knowledge and attitude of PTs regarding AI applications in rehabilitation based on multiple explanatory factors.

**Methods:**

A web-based Google Form survey, which was divided into 4 sections, was used to collect the data. A total of 317 PTs participated voluntarily in the study.

**Results:**

The PTs’ knowledge about AI applications in rehabilitation was lower than their knowledge about AI in general. We found a statistically significant difference in the PTs’ knowledge regarding AI applications in the rehabilitation field based on sex (odds ratio [OR] 2.43, 95% CI 1.53-3.87; *P*<.001). In addition, experience (OR 1.79, 95% CI 1.11-2.87; *P=*.02) and educational qualification (OR 1.68, 95% CI 1.05-2.70; *P*=.03) were found to be significant predictors of knowledge about AI applications. PTs who work in the nonacademic sector and who had <10 years of experience had positive attitudes regarding AI.

**Conclusions:**

AI technologies have been integrated into many physical therapy practices through the automation of clinical tasks. Therefore, PTs are encouraged to take advantage of the widespread development of AI technologies and enrich their knowledge about, and enhance their practice with, AI applications.

## Introduction

### Background

The use of artificial intelligence (AI) has been growing rapidly in the fields of health care and rehabilitation [1]. Many of AI’s clinical benefits have been mentioned in the literature. AI is defined as the ability of a machine to perform a functional task moderated intelligently by humans [2]. AI uses algorithms to learn, think, and then assist in various clinical practices such as radiology [3], dentistry [4], dermatology [5], and rehabilitation [6]. In addition, AI provides up-to-date clinical information from scientific resources such as journals, books, and evidence-based practice, which assists health care providers in clinical decision-making. Furthermore, AI technologies help to reduce medical errors in daily human practices [7-9].

Today, AI technologies are used in multidisciplinary health care research fields, and researchers are exploring and investigating the practical implications of using such technologies. In rehabilitation, AI has been used to enhance the patient care process by assisting physical therapists (PTs) either in providing a comprehensive assessment or in predicting patients’ performance or determining a diagnosis [10]. Moreover, research has revealed more uses of AI in medical and rehabilitation practices, such as problem solving, x-ray diagnosis, planning treatment protocols, and physical manipulation of patients [11]. All these functions of AI are core elements of physical therapy professional practice. Consequently, it is worth stating that many physical therapy practices might be susceptible to automation by AI technologies. In the study by Brougham and Haar [12], futurists are quoted as predicting that a third of the jobs that exist today could be taken by smart technology, artificial intelligence, robotics, and algorithms by 2025.

Machine learning (ML), a subset of AI, enables practitioners to use known quantities from data to make predictions [13]. In addition, ML is used to enable computerized decision-making and provide predictions based on patient data, and it can also be used as a tool to provide immediate preventive care for patients with specific conditions [14]. In 2020, a study was conducted by Ye et al [15] to validate a tool that was developed based on ML algorithms to predict older adults’ fall risk. The researchers found that the ML-based fall risk tool was a valid tool for producing automatic early warnings, which may prevent falls among older adults. In fact, patients with orthopedic and neurological disorders need an intensive rehabilitation physical therapy program that might last for months to improve their functional disabilities. Subsequently, PTs might face challenges in designing therapeutic interventions based on their understanding of the patients’ performance. In such cases, an ML-based AI decision support system would help PTs in determining diagnosis and monitoring the rehabilitation intervention.

By contrast, as AI technologies become more widespread, the need for AI education among PTs becomes essential. A qualitative study was conducted in the United Kingdom by Castagno and Khalifa in 2020 [2] to explore the knowledge and attitudes of health care providers regarding current and future uses of AI. The researchers reported a lack of full understanding of AI fundamentals as well as concerns about the potential consequences of the use of AI in clinical practice among health care professionals. Given the fact that AI technologies may perform some of the PTs’ work, it is necessary to urgently investigate PTs’ perception and preparation for using these advanced technologies. Understanding PTs’ perception would help in maximizing confidence in, and enhancing comfort regarding, the use of 21st century advanced technologies in physical therapy practices.

### Objectives

Because of the fast pace of innovation in AI and digital technologies, it is impossible to ignore the current debate about the importance of these technologies in clinical practice, especially in rehabilitation. However, we have to first set the stage for this digital revolution by ascertaining PTs’ knowledge and attitudes regarding this new era of health and rehabilitation practices. Although previous research has identified the various applications of AI in health care and rehabilitation [16], little has been investigated about PTs’ knowledge and attitudes regarding AI applications. Therefore, the aim of this study was to explore PTs’ understanding of the AI technologies used in health care and rehabilitation. In addition, this study assessed the relationship between PTs’ knowledge and multiple demographic variables, including sex, educational qualification, years of experience, workplace setting, and number of AI applications at work. The results of this study would help in filling the gap in current research recommendations as well as academic and clinical practices.

## Methods

### Participants

PTs were invited to voluntarily participate in the study. Only participants working in Saudi Arabia as licensed PTs could participate. In March 2021, a survey link was created using Google Forms (Google LLC). In the prefatory section of the survey, a brief description was provided to inform the participants about the goal of the study and to confirm the confidentiality and anonymity of their data. To obtain informed consent, a question about the participants’ agreement to participate in the study was placed at the beginning of the survey.

### Ethics Approval

Institutional review board approval was received from the Center of Excellence in Genomic Medicine Research (14-CEGMR-Bioeth-2021), approved by the National Committee of Bioethics, Jeddah, Saudi Arabia (KACST: HA-02-J-003).

### Instrument

The survey was developed through deep searching in the literature [2,12] and feedback from physical therapy experts. The face validity and content validity of the survey [17,18] were established by inviting 8 PTs who were experts in the field of rehabilitation and survey studies to review and rate each item of the survey for its appropriateness, clarity, ordering, and construct. Next, each expert’s comments were reviewed by the principal investigator to improve the quality of the survey questions and establish the content validity of the survey based upon 80% agreement of the experts’ feedback. The content validity index was 0.8 for the whole survey; however, the content validity index was between 0.8 and 1 for each item.

The survey, which was divided into 4 sections, consisted of 20 questions. The first section of the survey asked about the demographic characteristics of the participants to determine the sample age, sex, years of experience, educational qualification, number of AI applications at work, and subspecialization. The second section asked about participants’ knowledge about AI in the field of health care and rehabilitation. The third section sought participants’ opinions regarding the advantages and uses of AI as well as its impact on the future of rehabilitation. The final section concerned the ethical implications of using AI and participants’ willingness to explore the AI field. The answers to the survey questions were assessed using yes or no questions and a 5-point Likert scale (ranging from strongly agree to strongly disagree).

In this study, we investigated whether PTs’ knowledge and attitudes regarding AI medical applications differed depending on the respondent’s sex, years of experience, educational qualification, employment sector, and number of AI applications at work. For this study, years of experience were categorized as >10 years or <10 years. The work sector categories were nonacademic or academic. Educational qualification categories were undergraduate or postgraduate (master’s degree and PhD), whereas the number of AI applications at work was categorized as no AI application or at least one AI application.

### Procedures

The electronic open survey was distributed using social media, including WhatsApp, Facebook groups, and Twitter. In addition, contact was made with PTs via email with a request to forward the survey to other PTs if they knew one. The survey was open from March 2021 to May 2021. Before distributing the survey, the minimum sample size was calculated using G*Power (version 3.1; Heinrich Heine University) to achieve a power of 0.80. In the G*Power software, a logistic regression test was conducted for a priori power calculation with an odds ratio (OR) of 1.5 and significance level of .05. The minimum sample needed to achieve a power of 0.80 was 280 for our study. This indicates that the sample size attained in this study (n=317) was sufficient to detect an effect. The report of this study has been written according to the Checklist for Reporting Results of Internet E-Surveys guidelines [19].

### Statistical Analysis

After the data were collected, they were coded and entered into a spreadsheet using Microsoft Excel 2016. Data were analyzed using SPSS software (version 28.0; IBM Corp). Descriptive statistics were used to describe the sample’s demographic characteristics in frequencies and percentages. Chi-square tests and binary logistic regression analysis were used to investigate the differences in PTs’ perceptions regarding AI applications in health care and rehabilitation based on demographic characteristics. A *P* value of ≤.05 was considered statistically significant.

## Results

### Descriptive Statistics

A total of 317 PTs from different workplace settings participated in the study. The mean age of the participants was 33.38 (SD 7.31) years. With regard to the respondents’ sex, 52.4% (166/317) of the participants were male, and 47.6% (151/317) were female. Most (243/317, 76.7%) of the participants were working in nonacademic sectors, mainly at outpatient clinics and hospitals. Nearly half (152/317, 47.9%) of the participants were general PTs. The majority (193/317, 60.9%) of the respondents reported that they had not come across any AI application at work. Only a few (11/317, 3.5%) of the participants had been exposed to AI applications at work >4 times. Of the 317 respondents, 137 (43.2%) reported that they obtained information about AI primarily from social media, whereas 114 (36%) stated that they obtained information about AI primarily from articles and journals. Detailed demographic characteristics of the participants are presented in Table 1.

**Table 1:** Demographic characteristics of the participants, exposure to artificial intelligence (AI) applications at work, and sources of AI information (N=317).

Characteristics			Values
Age (years), mean (SD); median (range)			33.38 (7.31); 33 (22-63)
**Sex, n (%)**			
	Male	166 (52.4)	
	Female	151 (47.6)	
**Employment sector, n (%)**			
	Academic	74 (23.3)	
	Nonacademic	243 (76.7)	
**Educational qualification, n (%)**			
	Undergraduate degree	188 (59.3)	
	Postgraduate degree	129 (40.7)	
**Subspecialty, n (%)**			
	Cardiorespiratory	15 (4.7)	
	General	152 (47.9)	
	Geriatrics	8 (2.5)	
	Musculoskeletal and sports	102 (32.2)	
	Neurorehabilitation	23 (7.3)	
	Pediatrics rehabilitation	17 (5.4)	
**AI** **applications at work, n (%)**			
	0	194 (61.2)	
	1	56 (17.7)	
	2 to 4	56 (17.7)	
	>4	11 (3.5)	
**Source of** **AI** **information (multiple responses), n (%)**			
	Social media	137 (43.2)	
	Traditional media	50 (15.8)	
	Colleagues or friends	97 (30.6)	
	Class lectures	80 (25.2)	
	Articles or journals	114 (36)	
	Workshops	44 (13.9)	
	Work	37 (11.7)	
	Web-based courses	29 (9.1)	
	No prior information	36 (11.4)	

### Knowledge About AI

Table 2 shows the results of the binary logistic regression analysis to find the statistically significant differences in the PTs’ AI knowledge—general, health care, and rehabilitation—based on the demographic variables.

**Table 2:** Results of logistic regression analysis to assess the factors associated with artificial intelligence (AI; N=317).

Variable					B			95% CI for B			SE for B	β		*P* value
**Knowledge about AI in general**														
	**Sex**													
		Constant	1.48			N/A^a^			0.21			N/A		N/A
		Male	1.38			1.81-8.73			0.40			3.97		.001
		Female	Reference			N/A			N/A			N/A		N/A
	**Employment sector**													
		Constant	1.55			N/A			0.31			N/A		N/A
		Nonacademic	0.67			0.94-4.04			0.37			4.70		.07
		Academic	Reference			N/A			N/A			N/A		N/A
	**Experience (years)**													
		Constant	1.79			N/A			0.21			N/A		N/A
		>10	0.69			0.93-4.28			0.39			2.00		.08
		<10	Reference			N/A			N/A			N/A		N/A
	**Educational qualification**													
		Constant	1.838			N/A			0.21			N/A		N/A
		Postgraduate degree	0.54			0.82-3.62			0.38			1.72		.15
		Undergraduate degree	Reference			N/A			N/A			N/A		N/A
	**AI** **at workplace**													
		Constant	1.62			N/A			0.19			N/A		N/A
		≥1	1.54			1.76-12.32			N/A			4.66		.002
		0	Reference			N/A			N/A			N/A		N/A
**Knowledge about** **AI** **in health care**														
	**Sex**													
		Constant	0.50			N/A			0.17			N/A		N/A
		Male	1.38			2.28-6.92			0.28			3.97		<.001
		Female	Reference			N/A			N/A			N/A		N/A
	**Employment sector**													
		Constant	0.81			N/A			0.25			N/A		N/A
		Nonacademic	0.41			0.85-2.68			0.30			1.51		.16
		Academic	Reference			N/A			N/A			N/A		N/A
	**Experience (years)**													
		Constant	0.83			N/A			0.16			N/A		N/A
		>10	0.75			1.22-3.68			0.28			2.12		.008
		<10	Reference			N/A			N/A			N/A		N/A
	**Educational qualification**													
		Constant	0.78			N/A			0.16			N/A		N/A
		Postgraduate degree	0.91			1.41-4.40			0.30			2.50		.002
		Undergraduate degree	Reference			N/A			N/A			N/A		N/A
	**AI** **at workplace**													
		Constant	0.57			N/A			0.15			N/A		N/A
		≥1	1.96			3.41-14.97			0.37			7.15		<.001
		0	Reference			N/A			N/A			N/A		N/A
**Knowledge about** **AI** **in rehabilitation**														
	**Sex**													
		Constant		0.04			N/A			0.16			N/A	N/A
		Male		0.89			1.53-3.87			0.24			2.43	<.001
		Female		Reference			N/A			N/A			N/A	N/A
	**Employment sector**													
		Constant		0.27			N/A			0.24			N/A	N/A
		Nonacademic		0.28			0.78-2.24			0.27			1.32	.31
		Academic		Reference			N/A			N/A			N/A	N/A
	**Experience (years)**													
		Constant		0.26			N/A			0.15			N/A	N/A
		>10		0.58			1.11-2.87			0.24			1.79	.02
		<10		Reference			N/A			N/A			N/A	N/A
	**Educational qualification**													
		Constant		0.28			N/A			0.15			N/A	N/A
		Postgraduate degree		0.52			1.05-2.70			0.24			1.68	.03
		Undergraduate degree		Reference			N/A			N/A			N/A	N/A
	**AI** **at workplace**													
		Constant		–0.21			N/A			0.14			N/A	N/A
		≥1		2.26			5.13-17.87			0.32			9.57	<.001
		0		Reference			N/A			N/A			N/A	N/A

^a^N/A: not applicable.

### Knowledge About AI in General

#### Overview

Of the 317 PTs, 280 (88.3%) indicated that they had knowledge about AI in general. The data indicated that there were no statistically significant differences in AI general knowledge by employment sector, experience, or qualification. However, there was a significant difference by sex (OR 3.97, 95% CI 1.81-8.73; *P*=.001); that is, the male PTs were 3.97 times more knowledgeable about AI in general than the female PTs. In addition, this study found that the number of AI applications at work was a statistically significant predictor of AI general knowledge among PTs (OR 4.66, 95% CI 1.76-12.32; *P*=.002).

#### Knowledge About AI in Health Care

Of the 317 PTs, 238 (75.1%) indicated that they had knowledge about AI in the field of health care. In this study, employment sector was not a significant predictor of knowledge about AI in health care among the PTs. However, there was a significant difference in AI knowledge based on sex (OR 3.96, 95% CI 2.28-6.92; *P*<.001). Compared with the female PTs, the male PTs were 3.96 times more likely to be familiar with AI applications. Participants who had >10 years of experience were 2.12 times more knowledgeable about AI applications than those with less experience (*P*=.008). In addition, there was a significant difference in knowledge about AI in health care based on educational qualification (OR 2.5, 95% CI 1.41-4.40; *P*=.002). The results indicated that PTs with an undergraduate degree were 2.5 times less knowledgeable about AI applications than those with a postgraduate degree. Furthermore, the findings revealed that PTs who had experience of working with at least one AI application were 7.15 times more knowledgeable about AI health care technologies than those who had no experience of working with AI applications (*P*<.001).

#### Knowledge About AI in Rehabilitation

Of the 317 PTs, 121 (38.2%) reported that they had knowledge about AI applications in the rehabilitation field. The results showed that there was a statistically significant difference in PTs’ knowledge regarding AI in rehabilitation based on sex (OR 2.43, 95% CI 1.53-3.87; *P*<.001): the male PTs were 2.43 times more knowledgeable about AI use in rehabilitation than the female PTs. In addition, experience and educational qualification were significant predictors of knowledge about AI applications in rehabilitation among the PTs: OR 1.79, 95% CI 1.11-2.87; *P*=.02, and OR 1.68, 95% CI 1.05-2.70; *P*=.03, respectively. Moreover, the number of AI applications at work was a significant predictor of AI knowledge in rehabilitation (*P*<.001). The results implied that having worked with at least one AI application increases AI knowledge by 9.57 times compared with having no practical experience at work.

### Attitudes Regarding Advantages of AI

Using a 5-point Likert scale, participants indicated their level of agreement regarding three advantages of using AI applications in health care and rehabilitation: reducing therapist workload, prevention of diseases, and facilitating patient care. The participants’ levels of agreement (frequencies and percentages) regarding the advantages of using AI in clinical practice are detailed in Table 3.

**Table 3:** Participants’ attitudes regarding the advantages of using artificial intelligence (AI) in clinical practice (N=317).

Advantages of using AI in clinical practice and variables				Strongly agree		Agree		Neutral	Disagree		Strongly disagree
**Reducing therapist workload, n (%)**											
	**Sex**										
		Male	67 (21.1)		59 (18.6)		34 (10.7)		5 (1.6)	1 (0.3)	
		Female	41 (12.9)		68 (21.5)		36 (11.4)		6 (1.9)	0 (0)	
	**Employment sector**										
		Academic	23 (7.3)		35 (11)		12 (3.8)		3 (0.9)	1 (0.3)	
		Nonacademic	85 (26.8)		92 (29)		58 (18.3)		8 (2.5)	0 (0)	
	**Experience (years)**										
		>10	52 (16.4)		37 (11.7)		32 (10.1)		7 (2.2)	1 (0.3)	
		<10	56 (17.7)		90 (28.4)		38 (12)		4 (1.3)	0 (0)	
	**Educational qualification**										
		Postgraduate degree	55 (17.4)		45 (14.2)		23 (7.3)		5 (1.6)	1 (0.3)	
		Undergraduate degree	53 (16.7)		82 (25.9)		47 (14.8)		6 (1.9)	0 (0)	
**Facilitating patient care, n (%)**											
	**Sex**										
		Male	65 (20.5)		77 (24.3)		20 (6.3)		2 (0.6)	2 (0.6)	
		Female	43 (13.6)		78 (24.6)		25 (7.9)		5 (1.6)	0 (0)	
	**Employment sector**										
		Academic	26 (8.2)		36 (11.4)		9 (2.8)		3 (0.9)	0 (0)	
		Nonacademic	82 (25.9)		119 (37.5)		36 (11.4)		4 (1.3)	2 (0.6)	
	**Experience (years)**										
		>10	44 (13.9)		61 (19.2)		21 (6.6)		3 (0.9)	0 (0)	
		<10	64 (20.2)		94 (29.7)		24 (7.6)		4 (1.3)	2 (0.6)	
	**Educational qualification**										
		Postgraduate degree	42 (13.2)		68 (21.5)		14 (4.4)		3 (0.9)	2 (0.6)	
		Undergraduate degree	66 (20.8)		87 (27.4)		31 (9.8)		4 (1.3)	0 (0)	
**Prevention of diseases, n (%)**											
	**Sex**										
		Male	51 (16.1)		49 (15.5)		53 (16.7)		10 (3.2)	3 (0.9)	
		Female	31 (9.8)		52 (16.4)		53 (16.7)		13 (4.1)	2 (0.6)	
	**Employment sector**										
		Academic	27 (8.5)		25 (7.9)		17 (5.4)		5 (1.6)	0 (0)	
		Nonacademic	55 (17.4)		76 (24)		89 (28.1)		18 (5.7)	5 (1.6)	
	**Experience (years)**										
		>10	32 (10.1)		35 (11)		51 (16.1)		8 (2.5)	3 (0.9)	
		<10	50 (15.8)		66 (20.8)		55 (17.4)		15 (4.7)	2 (0.6)	
	**Educational qualification**										
		Postgraduate degree	39 (12.3)		35 (11)		41 (12.9)		11 (3.5)	3 (0.9)	
		Undergraduate degree	43 (13.6)		66 (20.8)		65 (20.5)		12 (3.8)	2 (0.6)	

#### Reducing Therapist Workload

More male PTs (126/317, 39.7%) than female PTs (121/317, 38.2%) agreed or strongly agreed that using AI reduces therapist workload. Moreover, a high percentage of the nonacademic participants (177/243, 72.8%) agreed or strongly agreed that using AI reduces the workload of PTs in clinical practice, whereas 77.7% (146/188) of the participants with <10 years of experience agreed or strongly agreed that using AI reduces the workload in PTs’ clinical practice. However, on the basis of educational qualification, a few participants (12/317, 3.8%) disagreed or strongly disagreed that AI is useful in reducing PTs’ workload.

#### Facilitating Patient Care

Of the 317 participants, more male PTs (n=142, 44.8%) reported their agreement that AI applications have advantages in facilitating patient care than female PTs (n=121, 38.2%). Of the 243 participants working in the nonacademic sector, most (n=201, 82.7%) agreed or strongly agreed that AI technologies can facilitate patient care in clinical practice. With regard to educational qualification, 81.4% (153/188) of the participants who had an undergraduate degree agreed or strongly agreed that using AI facilitated patient care in clinical settings. On the basis of years of experience, only 2.8% (9/317) of the participants disagreed or strongly disagreed that AI would be useful in facilitating patient care.

#### Prevention of Diseases

Of the 317 participants, more male PTs (n=100, 31.5%) reported positive attitudes regarding the advantage of AI technologies in preventing diseases than female PTs (n=83, 26.2%). In addition, of the 243 participants working in nonacademic sectors, 131 (53.9%) indicated that AI applications have a role in preventing diseases, whereas of the 74 participants working in academic organizations, 52 (70%) indicated that AI applications have a role in preventing diseases. In addition, the study results showed that of the 188 PTs with <10 years of experience, 116 (61.7%) had positive attitudes regarding using AI technologies to prevent diseases. Furthermore, participants with an undergraduate degree had a slightly higher level of agreement regarding the usefulness of AI technologies in preventing diseases than those with a postgraduate degree (109/188, 58%, vs 74/129, 57.3%, respectively).

### Uses of AI

Participants were asked to indicate their level of agreement regarding five aspects of the uses of AI: disease prediction, goal setting, assistive technologies, diagnostic tool, and education enhancement. Table 4 shows in detail the attitudes of the PTs regarding the uses of AI in clinical settings.

**Table 4:** Participants’ attitudes regarding the uses of artificial intelligence (AI) in clinical practice (N=317).

Uses of AI in clinical practice and variables				Strongly agree		Agree		Neutral		Disagree		Strongly disagree
**Disease prediction, n (%)**												
	**Sex**											
		Male	41 (12.9)		68 (21.5)		49 (15.5)		5 (1.6)		3 (0.9)	
		Female	20 (6.3)		53 (16.7)		63 (19.9)		12 (3.8)		3 (0.9)	
	**Employment sector**											
		Academic	17 (5.4)		28 (8.8)		27 (8.5)		1 (0.3)		1 (0.3)	
		Nonacademic	44 (13.9)		93 (29.3)		85 (26.8)		16 (5)		5 (1.6)	
	**Experience (years)**											
		>10	21 (6.6)		63 (19.9)		39 (12.3)		5 (1.6)		1 (0.3)	
		<10	40 (12.6)		58 (18.3)		73 (23)		12 (3.8)		5 (1.6)	
	**Educational qualification**											
		Postgraduate degree	32 (10.1)		48 (15.1)		41 (12.9)		5 (1.6)		3 (0.9)	
		Undergraduate degree	29 (9.1)		73 (23)		71 (22.4)		12 (3.8)		3 (0.9)	
**Goal setting, n (%)**												
	**Sex**											
		Male	44 (13.9)		74 (23.3)		22 (6.9)		24 (7.6)		2 (0.6)	
		Female	23 (7.3)		63 (19.9)		48 (15.1)		14 (4.4)		3 (0.9)	
	**Employment sector**											
		Academic	20 (6.3)		32 (10.1)		20 (6.3)		2 (0.6)		0 (0)	
		Nonacademic	47 (14.8)		105 (33.1)		50 (15.8)		36 (11.4)		5 (1.6)	
	**Experience (years)**											
		>10	26 (8.2)		60 (18.9)		24 (7.6)		19 (6)		0 (0)	
		<10	41 (12.9)		77 (24.3)		46 (14.5)		9 (6)		5 (1.6)	
	**Educational qualification**											
		Postgraduate degree	32 (10.1)		58 (18.3)		15 (4.7)		22 (6.9)		2 (0.6)	
		Undergraduate degree	35 (11)		79 (24.9)		55 (17.4)		16 (5)		3 (0.9)	
**Assistive technologies, n (%)**												
	**Sex**											
		Male	64 (20.2)		88 (27.8)		12 (3.8)		2 (0.6)		0 (0)	
		Female	55 (17.4)		70 (22.1)		25 (7.9)		1 (0.3)		0 (0)	
	**Employment sector**											
		Academic	22 (6.9)		41 (12.9)		10 (3.2)		1 (0.3)		0 (0)	
		Nonacademic	97 (30.6)		117 (36.9)		27 (8.5)		2 (0.6)		0 (0)	
	**Experience (years)**											
		>10	40 (12.6)		75 (23.7)		13 (4.1)		1 (0.3)		0 (0)	
		<10	79 (24.9)		83 (26.2)		24 (7.6)		2 (0.6)		0 (0)	
	**Educational qualification**											
		Postgraduate degree	42 (13.2)		78 (24.6)		8 (2.5)		1 (0.3)		0 (0)	
		Undergraduate degree	77 (24.3)		80 (25.2)		29 (9.1)		2 (0.6)		0 (0)	
**Diagnostic tool, n (%)**												
	**Sex**											
		Male	49 (15.5)		78 (24.6)		23 (7.3)		11 (3.5)		5 (1.6)	
		Female	32 (10.1)		64 (20.2)		43 (13.6)		10 (3.2)		2 (0.6)	
	**Employment sector**											
		Academic	24 (7.6)		33 (10.4)		11 (3.5)		6 (1.9)		0 (0)	
		Nonacademic	57 (18)		109 (34.4)		55 (17.4)		15 (4.7)		7 (2.2)	
	**Experience (years)**											
		>10	31 (9.8)		73 (23)		22 (6.6)		3 (0.9)		0 (0)	
		<10	50 (15.8)		69 (21.8)		44 (13.9)		18 (5.7)		7 (2.2)	
	**Educational qualification**											
		Postgraduate degree	36 (11.4)		67 (21.1)		14 (4.4)		9 (2.8)		3 (0.9)	
		Undergraduate degree	45 (14.2)		75 (23.7)		52 (16.4)		12 (3.8)		4 (1.3)	
**Education enhancement, n (%)**												
	**Sex**											
		Male	75 (23.7)		73 (23.1)		12 (3.8)		4 (1.3)		2 (0.6)	
		Female	54 (17)		66 (20.8)		29 (9.1)		2 (0.6)		0 (0)	
	**Employment sector**											
		Academic	33 (10.4)		34 (10.7)		6 (1.9)		1 (0.3)		0 (0)	
		Nonacademic	96 (30.3)		105 (33.1)		35 (11)		5 (1.6)		2 (0.6)	
	**Experience (years)**											
		>10	47 (14.8)		71 (22.4)		9 (2.8)		2 (0.6)		0 (0)	
		<10	82 (25.9)		68 (21.5)		32 (10.1)		4 (1.3)		2 (0.6)	
	**Educational qualification**											
		Postgraduate degree	59 (18.6)		62 (19.6)		5 (1.6)		1 (0.3)		2 (0.6)	
		Undergraduate degree	70 (22.1)		77 (24.3)		36 (11.4)		5 (1.6)		0 (0)	

#### Disease Prediction

Of the 166 male PTs, 109 (65.7%) agreed or strongly agreed that disease prediction is one of the uses of AI applications in clinical settings. In addition, 56.4% (137/243) of the participants working in nonacademic settings reported their agreement regarding using AI technologies in disease prediction. Of the 188 participants with <10 years of experience, 98 (52.1%) agreed or strongly agreed that disease prediction can be provided by AI technologies. However, on the basis of educational qualification, 7.2% (23/317) of the participants disagreed or strongly disagreed that AI could be used for predicting diseases.

#### Goal Setting

Of the 166 male participants, 118 (71.1%) agreed or strongly agreed regarding using AI applications for goal setting, whereas only 26 (15.7%) disagreed or strongly disagreed with the statement. Of the 74 participants working in academic organizations, only 2 (3%) disagreed or strongly disagreed that AI can be used for goal-setting purposes. With regard to years of experience, the majority (204/317, 64.4%) of the participants agreed or strongly agreed that goal setting could be facilitated by AI technologies. Similarly, on the basis of educational qualification, the majority (204/317, 64.4%) of the PTs agreed or strongly agreed that goal setting could be facilitated by AI.

#### Assistive Technologies

Of the 317 participants, 277 (87.4%) agreed or strongly agreed that AI applications can be used as assistive technologies in health care and rehabilitation. However, the male PTs (152/166, 91.6%) had a higher level of agreement than the female PTs (125/151, 82.8%). The results indicated that of the 243 participants working in the nonacademic sector, 214 (88.1%) agreed or strongly agreed that AI applications are among the assistive technologies used in the medical field. On the basis of experience and educational qualification, very few (3/317, 0.9%, in each category) of the participants disagreed about using AI applications as assistive technologies in health care.

#### Diagnostic Tool

Of the 166 male PTs, 127 (76.5%) agreed or strongly agreed that AI applications can be used to determine patients’ diagnoses. The majority (166/243, 68.3%) of the participants working in the nonacademic sector indicated that AI may help clinicians in providing medical diagnoses. In addition, we found that 63.8% (120/188) of the PTs with an undergraduate degree agreed or strongly agreed that AI technologies could be used for diagnostic purposes compared with 57.9% (73/129) of those with a postgraduate degree.

#### Education Enhancement

Of the 166 male participants, 148 (89.2%) agreed or strongly agreed about using AI technologies to enhance education among health care providers. In addition, our results revealed that of the 243 participants working in the nonacademic sector, 210 (86.4%) highly supported using AI technologies for education enhancement in the medical field. On the basis of experience and education, very few (2/317, 0.6%, in each category) of the participants strongly disagreed that AI has a role in enhancing the educational background of practitioners.

### Impact of AI

Participants were asked to indicate their level of agreement regarding 3 impacts of using AI technologies in health care and rehabilitation. The detailed results are presented in Table 5.

**Table 5:** Participants’ attitudes regarding the impact of artificial intelligence (AI) on the rehabilitation field (N=317).

The impact of AI and variables			Strongly agree		Agree		Neutral		Disagree		Strongly disagree
**Reducing human resources, n (%)**											
	**Sex**										
		Male	57 (18)	49 (15.5)		23 (10.1)		25 (7.9)		3 (0.9)	
		Female	27 (8.5)	55 (17.4)		50 (15.8)		15 (4.7)		4 (1.3)	
	**Employment sector**										
		Academic	15 (4.7)	30 (9.5)		20 (6.3)		8 (2.5)		1 (0.3)	
		Nonacademic	69 (21.8)	74 (23.3)		62 (19.6)		32 (10.1)		6 (1.9)	
	**Experience (years)**										
		>10	43 (13.6)	41 (12.9)		25 (7.9)		19 (6)		1 (0.3)	
		<10	41 (12.9)	63 (19.9)		57 (18)		21 (6.6)		6 (1.9)	
	**Educational qualification**										
		Postgraduate degree	50 (15.8)	34 (10.7)		26 (8.2)		17 (5.4)		2 (0.6)	
		Undergraduate degree	34 (10.7)	70 (22.1)		56 (17.7)		23 (7.3)		5 (1.6)	
**Increasing productivity, n (%)**											
	**Sex**										
		Male	52 (16.4)	86 (27.1)		23 (7.3)		3 (0.9)		2 (0.6)	
		Female	40 (12.6)	70 (22.1)		34 (10.7)		5 (1.6)		2 (0.6)	
	**Employment sector**										
		Academic	20 (6.3)	37 (11.7)		14 (4.4)		3 (0.9)		0 (0)	
		Nonacademic	72 (22.7)	119 (37.5)		43 (13.6)		5 (1.6)		4 (1.3)	
	**Experience (years)**										
		>10	32 (10.1)	73 (23)		21 (6.6)		3 (0.9)		0 (0)	
		<10	60 (18.9)	83 (26.2)		36 (11.4)		5 (1.6)		1 (1.3)	
	**Educational qualification**										
		Postgraduate degree	37 (11.7)	66 (20.8)		22 (6.9)		2 (0.6)		2 (0.6)	
		Undergraduate degree	55 (17.4)	90 (28.4)		35 (11)		6 (1.9)		2 (0.6)	
**Improving patients’ quality of life, n (%)**											
	**Sex**										
		Male	60 (18.9)	65 (20.5)		34 (10.7)		5 (1.6)		2 (0.6)	
		Female	42 (13.2)	65 (20.5)		36 (11.4)		5 (1.6)		3 (0.9)	
	**Employment sector**										
		Academic	24 (7.6)	33 (10.4)		14 (4.4)		3 (0.9)		0 (0)	
		Nonacademic	78 (24.6)	97 (30.6)		56 (17.7)		7 (2.2)		5 (1.6)	
	**Experience (years)**										
		>10	38 (12)	52 (16.4)		33 (10.4)		6 (1.9)		0 (0)	
		<10	64 (20.2)	78 (24.6)		37 (11.7)		4 (1.3)		5 (1.6)	
	**Educational qualification**										
		Postgraduate degree	44 (13.9)	48 (15.1)		30 (9.5)		5 (1.6)		2 (0.6)	
		Undergraduate degree	58 (18.3)	82 (25.9)		40 (12.6)		5 (1.6)		3 (0.9)	

#### Reducing Human Resources

Of the 166 male participants, 106 (63.9%) agreed that AI use has an impact on human resource reduction. In addition, we found that 58.8% (143/243) of the participants working in the nonacademic sector were highly of the opinion that the use of AI technologies may result in the reduction of human resources in the clinical field. Of the 188 PTs with <10 years of experience, 104 (55.3%) agreed or strongly agreed that AI use may result in human resource reduction. The results also showed that the participants with a postgraduate degree had a higher level of strong agreement about the impact of AI use on human resource reduction than those with an undergraduate degree (50/129, 38.8%, vs 34/188, 18.1%, respectively).

#### Increasing Productivity

The results showed that the majority (248/317, 78.2%) of the participants agreed or strongly agreed that work productivity could be increased by implementing AI in health care. Of the 243 participants working in the nonacademic sector, 191 (78.6%) agreed or strongly agreed that AI use could increase productivity. Very few (3/129, 2.3%) of the participants with >10 years of experience disagreed that an increase in productivity could be achieved by using AI technologies in health care. The PTs with an undergraduate degree had a slightly lower level of agreement on the role of AI in improving work productivity than those with a postgraduate degree (145/188, 77.1%, vs 103/129, 79.8%, respectively).

#### Improving Patients’ Quality of Life

The study found that more male participants (125/166, 75.3%) than female participants (107/151, 70.9%) agreed or strongly agreed that patients’ quality of life can be improved by using AI technologies in health care and rehabilitation. In addition, the study results indicated that 72% (175/243) of the participants working in the nonacademic sector significantly agreed that AI has a positive impact on patients’ quality of life. Furthermore, 75.5% (142/188) of the participants with <10 years of experience agreed or strongly agreed that using AI has a positive impact on patients’ quality of life.

### Ethical Implications of Using AI and Willingness to Explore the AI Field

The study investigated PTs’ ethical concerns that might arise when implementing AI in health care and rehabilitation settings. Nearly half (144/317, 45.4%) of the participants expressed concerns about the inability of AI applications to sympathize with human beings or understand the complexity of the human experience, whereas 42.9% (136/317) were concerned about the inability of AI applications used in health care to provide a judgment in unpredicted situations that are beyond the scope of the AI program. In addition, a few (36/317, 11.4%) of the respondents stated that they were concerned about AI developers not being from the medical field or having minimal experience in medical or clinical practice.

In addition, in response to the question “If the clinician’s judgment clashed with that of the AI application, which one should be trusted?” only 6% (19/317) of the participants stated that the AI application’s decision should be trusted. Most (262/317, 82.6%) of the PTs reported that the clinician’s judgment should be preferred over that of the AI application, whereas 11% (35/317) of the respondents expressed a preference for abiding by the patient’s choice when the clinician’s reasoning conflicted with the AI application’s decision. However, in response to a question about whether AI courses should be included in rehabilitation curricula, 71.9% (228/317) of the PTs responded in the positive.

## Discussion

### Principal Findings

The main purpose of this study was to obtain a snapshot of the overall perceptions and attitudes of PTs regarding AI applications in health care and rehabilitation. This study assessed the relationships among multiple factors, including sex, experience, employment sector, and educational qualification. To the best of our knowledge, this is the first study that examines PTs’ thoughts and opinions regarding AI technologies and their relationships with multiple explanatory variables. The study findings might add to the existing knowledge regarding why it is important to enhance PTs’ awareness of the advantages and uses of AI technologies in clinical practice.

In this study, it was found that the majority (health care: 238/317, 75.1%, and rehabilitation: 121/317, 38.2%) of the participants had moderate knowledge about AI in health care and rehabilitation. Most (196/317, 61.8%) of the respondents stated that they had not heard about AI applications in rehabilitation. The results were consistent with those of a study that was conducted in Canada to explore the perceptions of oncologists, physicists, and radiation therapists about AI, which reported moderate knowledge about AI applications in medicine [20]. In addition, similar findings were reported in an Australian study that highlighted the average knowledge about the impact of AI among different health care professions [21]. Surprisingly, the majority (194/317, 61.2%) of the respondents in this study reported that they had not come across any AI applications at their workplace. Although AI technologies have been a focus of medical research, real-world clinical practice still faces obstacles when it comes to implementing AI. To successfully implement AI technologies in rehabilitation, PTs need to have prior knowledge, practical experience, confidence, and acceptance of AI technologies. This study did not investigate the barriers to AI implementation in clinical practice; therefore, research could be conducted in the future to support this study’s findings.

Generally, the male participants reported having more knowledge and more positive attitudes regarding AI applications than the female participants. Similar findings were reported by Santos et al [22] who found that male students were more interested than female students in AI and robotics. Moreover, most (223/317, 70.3%) of the PTs expressed the view that AI applications would have an impact on health care and rehabilitation practices. However, participants with <10 years of experience were more likely to believe that AI would have an impact on clinical practice. This was consistent with the results of a previous study by Scheetz et al [21], which indicated that health care practitioners with fewer years of clinical practice, including ophthalmologists, radiation oncologists, and dermatologists, agreed that AI would have an impact on the workforce. The reasons behind this have not been investigated previously. However, it is possible, as noted in the results, that clinicians with more experience have less confidence in AI.

In this study, most (218/317, 68.8%) of the participants stated that they believed that AI would reduce PTs’ workload and increase their productivity. This finding was similar to those of studies of AI use among other clinicians [20,23]. However, employment sector was one of the explanatory factors in this study. We found that there was a statistically significant difference in the PTs’ responses based on their primary workplace. Participants working in the nonacademic sector were more likely to accept AI applications than PTs who worked in the academic sector. There are no prior studies on the differences in PTs’ knowledge and attitudes regarding AI based on their primary practice setting; therefore, the explanation is not clear, and more research needs to be conducted in this area. It is essential to have a better understanding of physical therapy educators’ knowledge and practical experience of, as well as confidence in, AI technologies because they are among the facilitators who would increase the acceptance of AI applications by future PTs.

Incorporating AI technologies in the physical therapy core curriculum would help to smoothen future PTs’ engagement with the new era of intelligent technologies in rehabilitation practices. Future PTs need to be mentally prepared to explore, understand, and apply the algorithms of AI applications in their practice. In this study, 71.9% (228/317) of the participants indicated that AI courses should be incorporated in the academic curriculum. Previous studies also suggested integrating different courses related to AI into undergraduate and postgraduate programs such as data science, deep learning, and behavioral science, which may help clinicians to understand and apply AI in their medical practice [6,24].

In addition, the results of this study indicated that only 6% (19/317) of the PTs think that the AI application’s decision should be preferred over that of the clinician, whereas the majority (262/317, 82.6%) of the PTs stated that they would abide by the clinician’s decision. A similar result was reported in the study by Oh et al [25], who found that the majority of the doctors would favor trusting their own opinion over that of the AI application when there was a difference of opinion. In this study, the results indicated that there is insufficient information about PTs’ knowledge of, and experience with, AI applications, especially in the rehabilitation field. This paper promotes the necessity for more research to be conducted to increase the knowledge and practical experience of PTs regarding AI applications.

This study includes some limitations. First, because the survey was self-administered, there is a possibility of some bias regarding the PTs’ responses. In addition, the results cannot be generalized to other health care professionals because this study was limited to PTs. In this study, an electronic survey was used to collect the data, and this may have led to sample selection bias. Other sampling strategies could be used in the future to reach out to a more representative sample of PTs. In physical therapy research, AI applications are being developed rapidly, but a very limited number of AI techniques are being implemented and translated into physical therapy practices. This study’s results indicate low-to-average AI knowledge among PTs and positive attitudes regarding the different advantages, uses, and impacts of AI use. However, action is required to translate AI technologies from research into actual clinical practice.

### Conclusions

The use of AI technologies is growing rapidly in health care and rehabilitation. Thus, there is a need to increase PTs’ awareness of various AI applications in rehabilitation to provide competent patient care facilities. The results of this study indicate that being a man, having >10 years of experience, and having a postgraduate degree are the anticipated PT criteria that increase AI knowledge and adoption levels. In addition, the results highlighted the importance of promoting evidence-based knowledge translation, particularly with regard to AI technologies, among PTs. However, to successfully implement AI in the rehabilitation field, further research on both physical therapy clinicians and patient expectations should be conducted.

## Abbreviations

AI: artificial intelligence
ML: machine learning
OR: odds ratio
PT: physical therapist
